# Erasmus+ Program and Nursing Students’ Sense of Coherence, Well-Being, and School Success

**DOI:** 10.3390/ijerph19073968

**Published:** 2022-03-26

**Authors:** Ivica Matić, Biljana Kurtović, Adriano Friganović, Cecilija Rotim

**Affiliations:** 1 School of Nursing Mlinarska, 10000 Zagreb, Croatia; ivica0909@gmail.com or; 2 Department of Nursing, University of Applied Health Sciences, 10000 Zagreb, Croatia; adriano@hdmsarist.hr (A.F.); cecilijarotim@gmail.com (C.R.); 3 Department of Anaesthesiology and Intensive Medicine, University Hospital Centre Zagreb, 10000 Zagreb, Croatia; 4 Rotim Polyclinic, 10000 Zagreb, Croatia; 5 Faculty of Dental Medicine and Health, Josip Juraj Strossmayer University of Osijek, 31000 Osijek, Croatia

**Keywords:** attitudes, Erasmus+ program, nursing students, school success, sense of coherence, well-being

## Abstract

Background: An international exchange program is an increasing phenomenon across Europe. Any opportunity for students to work or study abroad whilst undertaking their degree program could have a positive impact on their well-being, professional development, and general competences. Methods: The study was conducted on a cohort of 99 nursing students from the School of Nursing, who were followed before and after a one-month Erasmus+ mobility period. Quantitative data were collected using a sense of coherence, subjective well-being, and sociodemographic characteristics questionnaire. Qualitative data were collected using open-ended questions and interviewing the focus group. Results: Student mobility did not significantly alter their sense of coherence or subjective well-being. A significant positive correlation before and after mobility occurred between the sense of coherence, subjective well-being, and school success. According to the qualitative analysis, the students’ experiences were overall positive and stimulating, with many of them noticing better working conditions abroad. Conclusion: Although the international mobility of nursing students does not affect their sense of coherence and subjective well-being, it does contribute to changes in certain attitudes related to the profession and living conditions abroad. These findings may prove helpful in planning future mobilities during more favorable periods when school obligations are fewer.

## 1. Introduction

International youth mobility enabled by the Erasmus+ program is an important factor in the European promotion of education. It is believed that the program will continue to grow in importance as a fundamental form of youth internationalization [1]. This is supported by the fact that, in the last Erasmus + program (2014–2020), a total of 991 strategic partnerships and knowledge alliances were funded with the double objective of generating and diffusing innovation in higher education, as well as upgrading competencies across European national systems [2]. In Croatia, the availability of funds within the common budget for Erasmus+ programs increases each year. This also means that there is a growing number of financed projects and users who are given the opportunity to improve their knowledge, skills, and opportunities across the Europe [3]. Nursing students’ participation in Erasmus exchanges, as well as in different international programs, is considered an important learning occasion [4]. The results of other studies confirm that the Erasmus+ program is successful in other educational areas, such as pharmacy, and that it enables the exchange of concepts and relationships between students from different countries and professions and enriches their professional career prospects [5]. The success of the Erasmus+ program has also been confirmed in the undergraduate, master, and PhD students [5]. The experience of international mobility does not merely affect the development of professional competences, but also has an important influence on widening one’s possibilities [6]. Cultural knowledge can also be enhanced through intercultural encounters and engagement [7]. The main advantages of the program have been explored in numerous studies whose results have emphasized its positive aspects. Student mobility programs are strengthened by exposure to other cultures and their different lifestyles, languages, and health systems, which improves students’ educational abilities, as well as their experiences, communication skills, and interpersonal relationships [8]. Prior to the great expansion of the Erasmus+ program, Austin claimed that a program of international student mobility in the field of healthcare could improve students’ awareness of global differences in health and culture [9]. The program allows students to gain insight into new health systems, compare differences in providing nursing care, communicate with people speaking different languages, and explore new cultures [10]. However, there are certain negative connotations. Students often feel homesick, as well as a lack of integration in the initial stages of the mobility, while others develop the desire to leave their own countries under the influence of internationalization. However, if students do not develop the feeling of a loss of autonomy and manage to integrate into the new culture, their experiences are generally positive [10].

This paper presents the results of a study on the influence of international student mobility on the sense of coherence, subjective well-being, and school success, as well as changes in students’ attitudes toward their chosen profession and certain domains of life. We find this to be of great importance, because today’s youth will make changes in the nursing profession in the future, while new professional and personal experiences gained through the Erasmus+ program may affect their capacities for confrontation and their subjective well-being, as well as their knowledge and experience of nursing.

The sense of coherence (SOC), as a leading construct in health promotion, is a life orientation that an individual has about themselves and the world around them. It is defined as a “general orientation which expresses the extent to which a person has a dominant, persistent, yet dynamic sense of confidence that internal and external stimuli is structured, predictable, and explainable; that the power necessary to satisfy the demands of the stimuli is available; and that those demands are challenging and worthy of investment and effort” [11]. The role of the sense of coherence in the promotion of health and well-being, has been confirmed in numerous studies [12,13]. According to Antonovsky’s theory, individuals with a strong SOC are more resistant to stress because they perceive events as logical even if they are out of one’s control [11]. Such individuals prefer to perceive life events as explainable, as well as believe that they can control such events due to available resources, and that solving challenges is a valuable investment of effort [14]. The three basic components of the SOC are comprehensibility, manageability, and meaningfulness. Comprehensibility means that a person perceives stimuli as explainable, meaningful, and structured, rather than chaotic, inconsistent, or unexpected. Manageability involves confidence in the available powers or resources when facing demands. Meaningfulness is a motivational component related to an individual’s belief that life demands which we face are worthy of effort, and that it is valuable to dedicate oneself to them even when they are undesired [11]. This means adopting the habit of perceiving everyday life tensions as consistent, purposeful, and socially valuable. Some studies have found that gender significantly affects SOC [15,16], for example, that among female international students, a sense of coherence is positively related to the use of emotion-oriented strategies [15]. It was also found that SOC can be influenced by the socio-cultural characteristics of the country from which the students come [16].

One of numerous mechanisms for strengthening SOC involves the engagement of life experiences, such as travelling and internationalization. Therefore, students’ mobility could also positively affect their SOC. Strong levels of SOC in students are related to a greater motivation to study something desirable and students’ obtaining high academic performance, even though nursing is a demanding and high-stress profession [17]. The concept of a sense of coherence is suitable for application in schools and with young people, because it focuses on the sources and factors that strengthen health.

Science of subjective well-being has grown in the past decade, and it presents an actual research concept, especially concerning the adolescent period, which is less researched [12]. There are more theories and definitions describing SWB, but the most significant contribution was made by Diener who describes the concept as a multidimensional construct that includes long-term aspects, such as life satisfaction, combined with short-term aspects, such as a sense of happiness. Therefore, the term SWB is often used as a synonym for both happiness and life satisfaction. Diener was one of the first researchers who have summarized some of the SWB theories, emphasizing that life satisfaction or dissatisfaction appears because of the constant comparisons of one’s personal achievements with the achievements of others, along with personal desires [18]. This wide concept includes a person’s physical and psychological state as well as their social relationships, personal beliefs, and their degree of independence, all of which are rooted in a cultural, social, and environmental framework. When people reflect on their lives, they compare the standards they have for a good life. Higher subjective well-being has been associated with good health and longevity, as well as better social relationships, work performance, and creativity. Scientific studies estimate that 70% of SWB is attributable to environmental effects [19]. However, the research about SWB has been conducted in a Western industrialized and rich country, so we thought it was important to conduct the questionnaire in Croatian adolescents who do not live in a such country. It is essential to track citizens’ subjective senses of happiness and life satisfaction to improve societal conditions and maximize the fulfilment of human potential [19]. We assumed that travel, grants, and staying in a foreign country can increase adolescents’ SWB, as well as their motivation to live abroad and the improvement of their school success.

School success is often considered to be excellent achievement in the educational process, in terms of adopting educational goals and achieving good grades on exams during education. In our context, the variable which is most often equiponderated with school success is grades. The value of a student’s response is marked with the units of the school grade scale (from 1—unsatisfactory to 5—excellent), therefore assessing the student’s knowledge. Moreover, additional indicators, such as self-evaluation, evaluation by others, group work, project work, portfolios, etc., are frequently added to the assessment of one’s success. Students’ mobility provides an opportunity to include these different indicators of success assessment, not only in terms of cognitive and professional training, but also in terms of the improvement of other skills, such as cultural, language, and coping skills, along with living in a foreign country. The adolescents’ school success, in addition to personal success, is influenced by many factors from their environment, so we assumed that the international exchange program can affect the improvement of school performance.

Since it has been confirmed that the SOC contributes to mental health and well-being [20], the main priority of researchers in the field of adolescent sense of coherence is to explore the possible factors that strengthen the sense of coherence [21]. The aim of this study was to explore how the participation of students in international mobility affects their sense of coherence, subjective well-being, and school success and whether it can contribute to changes in the students’ attitudes regarding their future nursing profession and certain important life domains. In this paper, we hypothesized that international exchange programs will improve students’ sense of coherence, well-being, and school success. Moreover, we hypothesized that student attitudes on certain social issues in the context of mobility will be changed.

## 2. Materials and Methods

### 2.1. Study Design

A longitudinal cohort study was conducted on participants who took part in an Erasmus+ mobility program organized by the School of Nursing in Zagreb, Croatia. The school is an Erasmus+ certified institution that implements international mobility programs into its curriculum. That includes students’ selection, organizational support, and linguistic, professional, and cultural preparation. Mobility lasts for a month, and students undertake clinical internships in the field of nursing, full time, mentored by staff of the receiving institution. Upon returning to their country, students conduct dissemination activities aimed at expanding their experiences of acquired competences. The data was gathered two weeks before and two weeks after a one-month mobility in a European country. The SOC was measured by the Orientation to Life Questionnaire [11], which has been translated into more than 50 languages and used in more than a third of the nations of the world on all inhabited continents [22] and has also been translated and used with Croatian examinees [23]. The students marked their response to each of the 29 items on a seven-point scale with two anchoring responses. The total sum ranges from 29 to 203. A higher score indicates stronger SOC. The internal consistency of the scale was found to be high, with Cronbach’s α ranging from 0.82 to 0.95 [24]. The internal consistency in the present study showed Cronbach’s α was 0.87 before mobility and 0.90 after mobility. Subjective well-being (SWB) was measured by the Personal Wellbeing Index (PWI), translated and used on Croatian examinees [25]. The questionnaire consists of seven items in which examinees evaluate their own satisfaction with certain domains of life on an 11-point rating scale ranging from 0 (not satisfied at all) to 10 (extremely satisfied). All reported values were converted into a standard 0–100 range by shifting the decimal point one step to the right, where 70+ points indicates a normal level of SWB. According to the PWI scale, Cronbach’s α ranged from 0.70 to 0.85 [26]. The internal consistency in the present study showed Cronbach’s α was 0.91 before mobility and 0.89 after mobility. School success was measured based on the average of all grades received three months before starting mobility and the average of all grades received three months after returning from mobility. The data on school success were gathered using the electronic grade book system. Sociodemographic data included age, gender, mobility destination, and self-evaluated financial status. The participants evaluated their agreement with certain attitudes in the context of mobility by providing dichotomic answers (I agree, I disagree). The attitudes were the following: I plan to move abroad, Conditions abroad are better, The financial status of nurses abroad is better, The state system is better abroad, Education is better abroad, Subjective well-being is better abroad, People work harder abroad, Loneliness and nostalgia are more frequent abroad, and You are always a stranger abroad and it is difficult to fit in. Qualitative data was gathered using the same questionnaire with two written open-ended questions regarding expectations before and after mobility. The questionnaire contained one open-ended question without a time limit for answering. The question asked before mobility was: What are your expectations of staying abroad? After mobility, the question was: Did staying abroad meet your expectations? After the mobility period, the study was expanded to examine why the students’ success in school declined. The mini focus group was moderated by one of the researchers, who was also the Erasmus+ project coordinator in the school. The interviews employed the following question: What do you think could be the reason for your decline in school success after mobility?

### 2.2. Participants

After the school was awarded funds for the implementation of the Erasmus project by the European Commission, students were invited to send in applications for mobility during October 2019. Based on the motivation letter, curriculum vitae, language skills, regularity of attending classes, and school success, the participants who achieved the highest number of points were recruited for mobility, which was realized during January, and they agreed to participate in this research after entering the Erasmus project. The implementation of approved projects and monitoring of the regularity of spending allocated funds in Croatia are conducted by the Agency for Mobility and EU Programs. A cohort of 99 nursing students who were followed before and after a one-month Erasmus+ mobility period was of the average age of 18.57 ± 0.62, of whom 76% were female and 24% male. The students belonged to the same generation from the School of Nursing. Their financial status was evaluated as ‘average’ by 83% of the participants, while 17% of them evaluated it as ‘above average’. Most of the participants, 36%, stayed in Ireland, while 22% were in Germany, 21% in Portugal, and 20% in Malta (Table 1).

The qualitative study using content analysis is based on six participants whose school success suffered the most significant decline.

### 2.3. Data Analysis

Cronbach’s α was computed to estimate the internal consistency of instruments. The normality of variable distribution was tested using the Kolmogorov–Smirnov test. The values were given as mean (M) and standard deviation (SD), and differences between the groups were tested using a paired sample t-test. Differences in student attitudes about some social issues in the context of mobility were tested using the McNemar Bowker test. The correlation of the results of the questionnaire was determined using correlation analysis. *p*-values ≤ 0.05 were considered statistically significant. All analyses were performed in IBM SPSS Statistics for Windows, version 23.0 (IBM SPSS, NY, USA). Text deconstruction was conducted in the analysis of data received using the open-ended question regarding expectations before and after mobility. In the assessment of the problem of a decline in school success after mobility, qualitative content analysis was used [27].

### 2.4. Ethical Considerations

All participants were informed about the study’s aim before the trip in a meeting organized by the Erasmus+ coordinator. The students received a letter that explained the purpose of the study, emphasizing that participation was voluntary and anonymous and that the collected data would be used and published for scientific purposes. The participants signed an informed written consent.

## 3. Results

### 3.1. Participants’ SOC, SWB, and School Success

The participants’ SOC, SWB, and school success are presented in Table 2. No significant difference was found regarding the level of the sense of coherence before and after mobility. The distribution of the participants’ SOC has an average value of 140.19 ± 18.21 before mobility and 138.12 ± 21.99 after mobility. No significant difference was found regarding the components of the sense of coherence before and after mobility. Meaningfulness, as a motivating component of the sense of coherence, was the most prominent component among the participants. No significant difference was found in the SWB of the participants regarding international mobility. The participants’ average value of personal well-being was 82.10 ± 1.54% SM before mobility and 82.75 ± 1.52% SM after mobility. Satisfaction with one’s own safety was the component of SWB that received the best score. A significant difference was found regarding school success among participants before and after mobility. Three weeks after their return from mobility, the students had significantly lower grades than before mobility.

### 3.2. Correlations between SOC, SWB, and School Success

The Pearson correlation coefficient is presented in Table 3. Significant positive correlations were found between SOC and SWB before and after mobility (*p* < 0.001), as well as a significant positive correlation with school success before and after mobility (*p* < 0.001).

### 3.3. Student Attitudes on Certain Statements in the Context of International Mobility

Regarding the attitude related to the difficulty of fitting in abroad because one is always a foreigner, there was a significant change in the opinion of the participants before and after mobility. Of the 34 participants who agreed with the attitude before mobility, six of them changed their opinion after mobility. Regarding the attitude about a higher quality of life abroad, there was a significant change in the opinion of the participants before and after mobility. Of the 81 participants who believed that quality of life was better abroad before mobility, 17 of them changed their opinion after mobility. Regarding the attitude that people work harder abroad, there was a significant change in the opinion of the participants before and after mobility. Of a total of 48 participants who believed that people work harder before mobility, 24 of them changed their opinion after mobility (Table 4).

The experience of international mobility did not affect the participants’ beliefs about the sense of nostalgia while living abroad, emigrating from their country after finishing education, the difference in working conditions abroad, the quality of the state system, or the financial situation and education of nurses.

### 3.4. Changes in Expectations Regarding International Mobility of Students

The participants’ answers to the questions “What do you expect to achieve from your stay abroad?” can be grouped into several categories: achieving new experience, encountering new working conditions, learning about living conditions and culture, widening their circle of acquaintances, and improving communication skills in a foreign language.

Most of the student replies (36) refer to achieving new experiences. This general category includes answers such as: *new experience, positive and truly great experience, wonderful experience, wider horizons, better perspective on life*, etc. According to the number of replies (20), the second group is related to expectations of learning about working conditions in the country the students visited. For example, some of the answers were the following: *I am very interested in how nurses work there*; *I am interested in how people work in other countries*; *I want to learn about how they work and their working conditions*; *I want to see the working conditions of nurses and compare them with the conditions in Croatia*. Along with working conditions, some students (16) generally expected to learn about the living conditions and culture of the country where they were going to stay. Some of the replies from this category are related to the following expectations from their stay abroad: *learn about life in Ireland in detail*; *what to expect once I move there*; *learn about other cultures and their way of life*; *meet people and their customs and landmarks*; *I expect to be dissuaded of the notion that everything is that amazing abroad and that one can, in fact, live well in Croatia*; *I will try to understand their way of life*; *I am very interested in how nurses work there and what their educational system is like*. Furthermore, some students (18) expected to make new acquaintances (*make new acquaintances* and *meet new people*). Some students (14) stated that they expected that their stay abroad would improve their understanding of English, as well as their communication skills. Several students’ replies (12) related to their expectations regarding independence and resourcefulness (*greater independence*; *coping with staying with a different family; working in a new workplace and living in a new city; what it is like being separated from my family*).

Students’ replies to the question “What did you achieve from your stay abroad?” can be grouped into several categories: new experience, meeting a foreign culture, widening the circle of acquaintances; improving communication skills in a foreign language; personal growth.

Most of the student replies (32) to this question can be placed into the category of ‘new experience’. Some of the replies from this category are the following: *Ireland was a wonderful experience; I saw many things that are not available in Croatia; I gained a new experience, something unlike anything I had experienced before.* Students (20) also stated that their stay abroad brought them new acquaintances and/or friendships (*I met new people and made new friends in Ireland*, etc.).

Most of the students (20) stated that, during their stay abroad, they realized that the working conditions were better than in Croatia. Some of the replies were: *everybody knows their job*; *I believe things are much better in Ireland, nurses have less work and better salaries, and the working conditions are much better than in Croatia*; *Their healthcare system is much better*.

Several replies (16) related to a general improvement in communication and knowledge of English. For instance, students stated the following: *Interacting with other people in English was a pleasant experience; my knowledge of English improved.* The remaining replies (14) can be grouped under the category of ‘personal growth’. The students gave the following replies: *a feeling of satisfaction and fulfilment*, *more self-confidence*; *a better perspective on life*; *I have become more independent and open in communicating with people*; *I grew closer to my school colleagues*, *I found my way around successfully and managed to meet some of my goals*.

Some students (8) stated an improvement in their professional knowledge. They believe that the stay abroad contributed to their *knowledge in nursing care and enabled professional improvement*, that it brought them *even more knowledge and better competences*, enabled them to *learn about a different way of working*, as well as *expanded the horizons related to the nursing profession.*

Most of the students who commented on general living conditions (12) believed that the living conditions and financial situation are better abroad. Some of the replies were: *I think that life in Germany is much better because people are not under constant stress, as they are in Croatia, and that is why people are in a better mood and the entire atmosphere is calmer and better*; *They do not have to think about whether they can afford certain things.*

### 3.5. Factors That Have an Influence on the Participants’ School Success

Most of the students’ replies can be grouped into the category representing unreasonable expectation on the part of their teachers. The students believed that during their stay abroad their teachers expected them to study their subjects regularly and completely dedicate themselves to school while on mobility, as well as know the entire content of their subjects that was covered during their absence from school upon return. Here is one example of a reply: *Many teachers expected us to constantly study their subjects while abroad. So, they would send me presentations, and we were supposed to bring notebooks and all our books with us.* Student replies regarding their sense of a lack of understanding on the part of some teachers built upon the replies about unreasonable expectations. The students felt there was injustice, as well as a lack of understanding from the teachers regarding their stays abroad. Here are some examples of replies: *Many teachers had no understanding for our stay abroad; I tell him, “I wasn’t here for almost one month, I don’t see how you can expect me to know absolutely everything”, and then in the end of course I got a failing grade.* Students also stated that they experienced negative teacher behavior upon their return to school. They described such behavior as envy on the part of teachers. Here are some examples of replies: *It’s as if they are having their revenge because they couldn’t come with us*; *Extreme envy, everybody felt it.* Students also stated the problem of a lack of time for study during their stay abroad. They stated that they had an organized daily program, with very little free time, due to which they lacked time for study. Here is one reply as an example: *In fact, we had very little time. We had some free time around nine, after dinner. Maybe some free time in the afternoon, but only until dinner.*

## 4. Discussion

Many different benefits of implementing a short-term student mobility program have been highlighted in previous studies. Such benefits include cultural, personal, and employment outcomes [28,29,30,31], which are supported by the results of our study that student expectations were mostly justified and that the participation of nursing students in international mobility does not affect their sense of coherence nor their subjective well-being. Moreover, a sense of coherence, together with well-being, is a stable construct and a short period of mobility is probably not a sufficient stimulator to cause any significant changes. Although the author of the salutogenic theory, Antonovsky, claimed that the sense of coherence is intensively formed during childhood and the adolescent period and stabilized around 30th year of life, the authors of some additional longitudinal research have found a stable SOC during adolescent period [32,33]. Considering the results found in this research, we assume it is necessary to conduct more longitudinal studies to revise theoretical facts concerning the stability and the development of SOC, since it is indicative that SOC is stable at a much earlier age than adolescence [32]. The theoretical assumption of SWB stability has been empirically confirmed. By statistically processing the standardized results from other studies, Cummins determined the mean SWB score and its stability thanks to the homeostatic mechanism of each individual. Some events may reduce or increase life satisfaction, but the individual will return to a starting value of 75% SM [34] over a period of time. Obviously, in the short term, students’ mobility is not a significant event that changes the SWB. The positive correlation between the SOC and SWB found in our study extends the knowledge from previous findings [12]. The SOC seems to be a resource that enhances well-being by good perceived health. According to salutogenic theory, SOC is a factor promoting well-being, since all dimensions of well-being can be understood as general resources of resilience that strengthen SOC, therefore improving the quality of life. Moreover, an increased sense of coherence and subjective well-being before mobility has a significant connection with a greater sense of coherence and subjective well-being after mobility. Although some studies determine the diversity in SOC in relation to gender and the country from which students come [16], we believe that this should be further explored in future research also conducted in Croatia. International mobility contributed to a change in the attitudes of the participants regarding the difficulties of integrating in a foreign country, subjective well-being abroad, and the complexity of working abroad. This is supported by the result of our study on the positive correlation between SOC and SWB. Consequently, it is to be expected that a student who has the ability to deal with stress will learn more easily. Student expectations before mobility were confirmed regarding the factors of achieving new experience, widening their cultural competences, personal and professional improvement, and improvement of communication skills in a foreign language. Studies of students ’attitudes prior to mobility have shown that their main motives for going abroad are meeting new people, learning about a different culture, and personal development [35]. We claim that nursing students objectively learn about real situations abroad, because the diversity of approaches in resolving a specific situation can contribute to the development of students’ critical thinking and ultimately the selection of the best evidence-based practices. Studying abroad includes nursing skills which are under the supervision of a mentor in a real situation and which may be in a clinical environment or a practical skills cabinet with a simulated situation. This approach to learning nursing skills is applied at every level of nursing education, and student mobility cannot ultimately affect the qualification of a nurse.

A study conducted by Asoodar et al. showed that students’ motivation before leaving refers to beliefs that their upcoming stay abroad will be successful if it allows them to become more confident and independent [36]. Van der Beek and Van Aart collected data from a survey conducted on the “Student Experience Exchange” online platform, with 6,923 international students expressing their preferences for international mobility [37].

The results shows that students are generally very satisfied with the experience of studying abroad, and the factors that most affect student satisfaction are the new urban environment, study courses, professors, and a sense of the international environment. On the other hand, our findings suggest a significant reduction in student school achievement upon returning from mobility. We attribute this to the fact that educational content between countries is less similar than expected. In our study, students stated that, during their stay, they developed their relationships, improved their communication, and became more open to new experiences, but they also felt that they had too many school obligations, considering their everyday obligations. There is a question of how various measures in individual countries are targeted at the quality and substance of study programs (curricula) and what impact they have on teaching and learning processes [38]. The results of qualitative analysis emphasize the lack of time for studying theoretical content during mobility, the focus of which was on skills. Unreasonable expectations, a lack of understanding, and inappropriate behavior on the part of the teachers after mobility were the main factors in lower school success, which is mostly based on assessments of theoretical knowledge. Students find that their teachers’ expectations regarding school obligations were unreasonably high, and that they required time after their return from mobility to catch up. They felt some teachers were unjust and lacked understanding, and they believed that once students return to school after mobility, they should have been given a short period without oral or written exams, which would significantly help their return to school and their fulfilment of obligations. In that sense, there is a fear that the student sees themself as not committed to the obligations or that the teacher sees them as such. There are results of one study that speak in favor of this, where a part of the respondents did not decide on mobility for fear that it would turn out that they have no responsibility towards the existing educational obligations [8]. According to students ’complaints about teacher behavior, it would be necessary to collect data on how participants, legislators, and other stakeholders envision the Erasmus experience and its effects. This would make it possible to understand how the perspectives and benefits of Erasmus programs presented by promotional bodies often focus on certain aspects while omitting other participants [1], such as teachers in the country from which the student starts mobility. Therefore, we recommend that future research should also focus on the impressions of homeland teachers after the return of students from mobility to the homeland.

However, the study had some limitations. The sample of nursing students was convenient due to the researchers’ specific field of interest. Moreover, a further weakness of the study design was the lack of a control group. A group of students from that same year who were not participating in the exchange program could had been tested for the same parameters over the same period of time for comparison, which is suggested for future research.

## 5. Conclusions

Although an international exchange program for nursing students does not affect the students’ sense of coherence and subjective well-being, it does contribute to changes in certain attitudes related to the profession and living conditions abroad. Based on the results of qualitative analysis, we may conclude that the students viewed their stays abroad as positive experiences. It is necessary to carefully plan the period of students’ mobility and ensure that all the participants show a maximum level of awareness, especially the teachers, in order to make the period of transition through the context of internationalization easier for the students and to avoid difficulties in fulfilling school obligations after returning from abroad.

## Figures and Tables

**Table 1:** Sociodemographic characteristics of participants (*n* = 99).

Students’ Characteristic	*n* (%)
Gender	
Male	24 (24)
Female	75 (76)
Age	
18 y.	50 (51)
19 y.	42 (42)
20 y.	7 (7)
Socioeconomic status	
Middle	82 (83)
High	17 (17)
Mobility destination	
Portugal	21 (21)
Germany	22 (22)
Ireland	36 (36)
Malta	20 (20)

**Table 2:** Participants’ sense of coherence, subjective well-being, and school success (*n* = 99).

Variables	Before Mobility	After Mobility	t (df = 98)	*p*
Sense of coherence	140.19 ± 18.21	138.12 ± 21.99	1.298	0.197
comprehensibility	4.34 ± 0.77	4.31 ± 0.83	−0.013	0.990
manageability	5.02 ± 0.68	4.91 ± 0.85	1.526	0.130
meaningfulness	5.32 ± 0.83	5.20 ± 0.90	1.542	0.126
Overall subjective well-being	82.10 ± 1.54	82.75 ± 1.52	−0.705	0.483
satisfaction with the standard of living	81.01 ± 1.45	80.40 ± 1.27	0.529	0.555
satisfaction with health	83.54 ± 1.51	85.25 ± 1.29	−1.409	0.162
satisfaction with life achievements	80.81 ± 1.52	81.92 ± 1.47	−0.732	0.466
satisfaction with close relationships	84.04 ± 1.39	83.74 ± 1.41	0.23	0.819
satisfaction with personal safety	84.04 ± 1.58	85.05 ± 1.39	−0.752	0.454
satisfaction with community belonging	81.92 ± 1.47	82.12 ± 1.47	−0.149	0.882
satisfaction with future safety	79.39 ± 1.36	80.81 ± 1.37	0.957	0.341
School success	4.50 ± 0.63	4.29 ± 0.63	3.220	0.002

**Table 3:** Correlations among sense of coherence (SOC), subjective well-being (SWB), and school success (SS) before (1) and after mobility (2).

	SS 2	SOC 2	SWB 2
SS 1	0.449 **	−0.059	0.089
SOC 1	−0.083	703 **	0.420 **
SWB 1	−0.042	0.379 **	0.681 **

** *p* < 0.001.

**Table 4:** Participants’ attitudes regarding certain statements in the context of international mobility.

Students’ Attitudes	Pre-Mobility	Post-Mobility			*p*
		Yes	No	Total	
I plan to move abroad	Yes	30	9	39	1.000
	No	9	51	60	
	Total	39	60	99	
Conditions abroad are better	Yes	55	26	81	0.117
	No	15	3	18	
	Total	70	29	99	
The financial situation of nurses is better abroad	Yes	76	14	90	0.115
	No	6	3	9	
	Total	82	17	99	
The organization of the state system is better abroad	Yes	73	9	82	0.405
	No	14	3	17	
	Total	87	12	99	
Education is better abroad	Yes	36	15	51	1.000
	No	15	33	48	
	Total	51	48	99	
Quality of life is better abroad	Yes	64	17	81	0.035
	No	6	12	18	
	Total	70	29	99	
People work harder abroad	Yes	24	24	48	0.001
	No	5	46	51	
	Total	29	70	99	
Loneliness and nostalgia are more frequent abroad	Yes	58	8	66	0.383
	No	13	20	33	
	Total	71	28	99	
You are always a foreigner abroad and it is harder to fit in	Yes	28	6	34	0.035
	No	17	48	65	
	Total	45	54	99	

## Data Availability

The data and the questionnaires from this study are available upon request from the corresponding author.
